# Predicting MicroRNA Mediated Gene Regulation between Human and Viruses

**DOI:** 10.3390/cells7080100

**Published:** 2018-08-08

**Authors:** Xin Shu, Xinyuan Zang, Xiaoshuang Liu, Jie Yang, Jin Wang

**Affiliations:** The State Key Laboratory of Pharmaceutical Biotechnology and Jiangsu Engineering Research Center for MicroRNA Biology and Biotechnology, NJU Advanced Institute for Life Sciences (NAILS), School of Life Science, Nanjing University, Nanjing 210023, China; cpu_shuxin@126.com (X.S.); hsinring@foxmail.com (X.Z.); xsliunju@foxmail.com (X.L.)

**Keywords:** miRNA, virus, host, Cross-Kingdom, target prediction

## Abstract

MicroRNAs (miRNAs) mediate various biological processes by actively fine-tuning gene expression at the post-transcriptional level. With the identification of numerous human and viral miRNAs, growing evidence has indicated a common role of miRNAs in mediating the interactions between humans and viruses. However, there is only limited information about Cross-Kingdom miRNA target sites from studies. To facilitate an extensive investigation on the interplay among the gene regulatory networks of humans and viruses, we designed a prediction pipeline, mirTarP, that is suitable for miRNA target screening on the genome scale. By applying mirTarP, we constructed the database mirTar, which is a comprehensive miRNA target repository of bidirectional interspecies regulation between viruses and humans. To provide convenient downloading for users from both the molecular biology field and medical field, mirTar classifies viruses according to “ICTV viral category” and the “medical microbiology classification” on the web page. The mirTar database and mirTarP tool are freely available online.

## 1. Introduction

MicroRNAs (miRNAs) are a class of small (~24 nt), non-coding RNA molecules that play a critical role in fundamental cellular processes and many types of diseases. They negatively regulate gene expression by binding to the 3′-untranslated regions (3′UTR) of the target mRNAs in cells [1]. Recent studies have found that they are involved in viral infections and play a key role in the host–virus interaction network. Host miRNAs modulate the expression of viral genes by targeting on virus transcripts, while viruses encode miRNAs that protect them from the host’s antiviral response by acting on cellular mRNAs [2,3,4,5]. Skalsky et al. [5] reported a comprehensive survey of viral and cellular miRNA targetome in Epstein-Barr virus (EBV)-infected lymphoblastoid cell lines using photoactivatable ribonucleoside-enhanced crosslinking and immunoprecipitation (PAR-CLIP) and deep sequencing technique combined with bioinformatics. In this survey, over 500 target sites of EBV miRNAs on cellular transcripts were detected in addition to the cellular miRNA targets on virus. This result may imply that viral miRNAs have a similar mode of multiple targeting as cellular miRNAs. Although the detection of miRNA targets by high throughput techniques remains a big challenge, there has been growing interest in the role of miRNAs in host–virus interactions.

The virus miRNAs can target both the host genes and viral genes in order to contribute to the creation of a propagating environment in the host cell [2]. EBV-encoded miRNA miR-BHRF1-2-5p blocks Interleukin-1 (IL-1) signaling by directly targeting the IL-1 Receptor 1 (IL1R1) [6]. Hancock et al. [7] found that human Cytomegalovirus (HCMV) also uses its own miRNAs, miR-US5-1 and miR-UL112-3p, which bind to IkB kinase (IKK) complex components IKKα and IKKβ, in order to avoid the immune response of the host. Some viral miRNAs show sequence similarity with host miRNAs and thus, may take part in the conserved cellular gene regulation network [8]. In Kaposi’s sarcoma-associated herpesvirus (KSHV)-infected human cell line, Manzano et al. [9] identified that KSHV miR-K3+1 and miR-K3 share perfect and offset 5′ homology with cellular miR-23, respectively. KSHV miR-k12-11 is an ortholog of miR-155, which can inhibit the 3′UTR region of BACH-1 [10].

Host miRNAs were found to target the viral RNA transcripts to inhibit viral pathogenesis, which essentially involves being a defense against viral infections [3,4,5]. It was reported that human miRNA effectively restricts the accumulation of the retrovirus primate foamy virus type 1 (PFV-1) in human cells, which involves hsa-miR-32 inhibiting the proliferation of PFV-1 by targeting PFV-1 F11 sequence. However, PFV-1 also encodes a protein named Tas, which suppresses miRNA-directed functions in mammalian cells and displays Cross-Kingdom anti-silencing activities [4]. This new report focused on an EBV-encoded protein EBNA2, which subverts immune surveillance by downregulating miR-34a that targets an important immune checkpoint PD-L1 in lymphoma B cells [11]. Human liver-specific miRNA hsa-miR-122 can induce hepatitis C virus (HCV) replication by targeting the 5′-non-coding region (NCR) of the viral genome [3]. The human miRNAs let-7b and mir-199a target the 5′UTR of HCV to decrease viral replication [12,13]. Pedersen et al. [14] also found that the overexpression of miR-196 and miR-448 significantly reduced the replication of HCV as they target the NS5A coding region and core of the HCV genome, respectively.

These findings indicate a common role of miRNAs in mediating the diversified interactions between humans and viruses. A total of 2588 mature human miRNAs and 181 mature miRNAs of human-related viruses have been recruited in mirBase so far (release 21) [15]. To facilitate the extensive investigation on the interplay among the gene regulatory network of humans and viruses, computational tools and comprehensive miRNA target repositories pertaining to human–virus interactions is necessary. These resources could provide the researchers with an efficient approach and potential miRNA targets to facilitate the investigation of miRNA function and regulation mechanisms. In particular, in the era of omics when it is possible to obtain a complete set of molecular data of gene expression, prediction tools and database are essential for genome-scale or microbiome-scale data analysis and help to decipher the panorama of the gene regulation network of human–virus interplay. This will ultimately facilitate the discovery of new drug targets for viruses, including HIV [16], HCMV [7] and HCV [3].

MiRNAs suppress interspecies gene expressions by targeting the 3′-UTRs of mRNAs during the infection or antiviral processes. Although many algorithms [17,18,19,20,21] are available for miRNA target prediction, only a few of them can be directly used to predict the interspecies regulation between viruses and hosts [20]. Most of the tools were designed for intra-species application by predicting the miRNA targets on their own genome, such as TargetScan, PicTar, miRanda and DIANA-microT [18,22,23,24]. In this situation, the databases of Cross-Kingdom miRNA target sites were produced by using the multiple intra-species target prediction tools mentioned above, which may possibly create concerns regarding the methodology and thus, the accuracy.

ViTa [25] provides predicted targets of host miRNAs from humans, mice, rats and chickens (mirBase release 8.2), which are located on 2108 virus species from 23 families. VHoT [26] houses predictions of 271 viral miRNAs on six hosts, which are namely humans, mice, rats, rhesus monkeys and cows. VmiReg [27] contains predicted targets of 169 viral miRNAs (from 10 types of viruses) on humans. VIRmiRNA [28] provides experimentally validated viral miRNAs and their targets on human and other species. All of these databases provide information of interspecies miRNA targeting in one direction only, which include either the target of viral miRNAs on host genes or the target of host miRNAs on viruses. To investigate the complex and dynamic interactions between the gene regulatory network of humans and infectious viruses that are mediated by miRNAs, the database mirTar was constructed that provides a comprehensive miRNA target repository pertaining to 2588 human miRNAs (mirBase release 21) that target 386 genomes of human-related viruses as well as 181 viral miRNAs that target the human genome. The new computational pipeline that was specially designed for human–virus interspecies miRNA target prediction was presented.

## 2. The prediction Tool and Results

### 2.1. Data Collection

A total of 2588 mature human miRNAs and 181 mature viral miRNAs were downloaded from mirBase (Release 21). Human genome and virus genomes as well as their classification information and taxonomy annotation were obtained and organized from NCBI [29,30]. Meanwhile, the annotation of gene name and protein name pertaining to the mRNA transcripts were acquired from Ensemble [31]. A total of 386 human-related viral species were collected that are belong to 34 families and fall under the following 7 genome types: (1) Deltavirus; (2) dsDNA viruses, no RNA stage; (3) dsRNA viruses; (4) Retro-transcribing viruses; (5) ssDNA viruses; (6) ssRNA viruses; and (7) unclassified viruses [32]. These viruses are all human infections. Some of them (79/386) are common and medically important viral species as categorized in medical microbiology, which mostly cause diseases of the respiratory tract, gastrointestinal tract and liver.

### 2.2. The Prediction Tool

Mainstream miRNA target prediction tools were limited to intra-species applications as they were only capable of predicting the miRNA targets on their own genome. Thus, the databases of interspecies miRNA targets were produced by using a combination of these methods as an approach to improve the reliability of the prediction results. For example, ViTa applied miRanda and TargetScan to identify the host miRNA target sites in virus genomes [18,23]. VHot combined five miRNA target prediction tools, which were namely TargetScan, miRanda, RNAhybrid, DIANA-microT and PITA, to form its prediction engine [18,23,24,33,34]. VmiReg predicted targets of viral miRNAs by four established prediction programs, which were namely miRanda, TargetScan, RNAhybrid and PITA [18,23,33,34]. This approach may create problems in inter-species target prediction as the sequence specificity of intra-species miRNA-target interaction are included. In addition, most of these calculations are quite time-consuming and require huge processing resources for a genome-scale prediction. To find miRNA targets across different kingdoms, we designed a prediction pipeline, mirTarP, that directly seeks the potential miRNA target. This can produce results quickly and thus, is very suitable for miRNA target screening using large-scale calculations.

**Figure 1:**
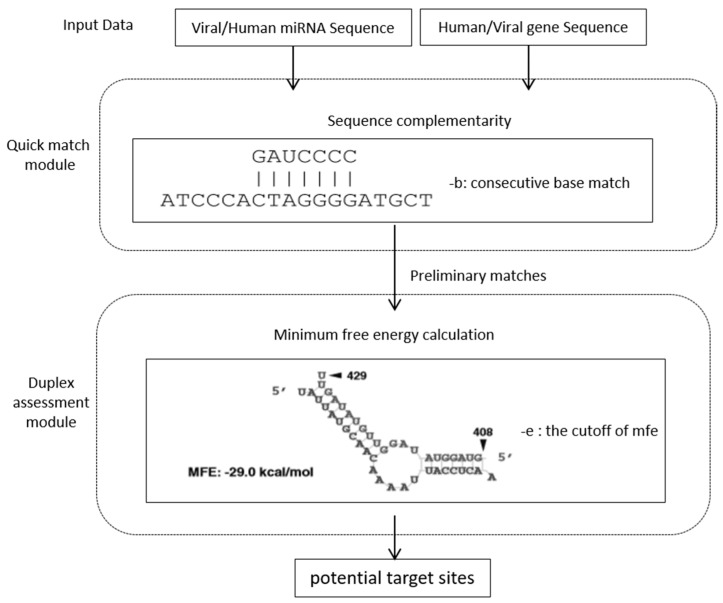
Flow chart of mirTarP for human–virus interspecies miRNA target prediction. The parameter ‘-b’ represents the number of consecutive base matches between miRNA and target sequence, while ‘-e’ represents the cutoff of minimum free energy of miRNA-target binding.

MirTarP was designed by integrating two classical algorithms of sequence analysis, which were Blast [35] and RNAhybrid [34]. They work as the cores of two modules included in mirTarP, which are quick match and duplex assessment. Blast uses heuristics to accelerate searches for similar segments of a sequence. A window of consecutive perfect match can be set when running the algorithm. To improve the calculation efficiency, mirTarP introduced the sequence similarity tool Blast to produce preliminary matches between the miRNA and its target mRNA sequences. The results from the quick match module were subsequently delivered to duplex assessment module, which uses the RNAhybrid program for the calculation of minimum free energy (mfe) of miRNA–mRNA hybridization duplexes based on the principles of thermodynamics. The mfe value stands for the stability of miRNA binding. To assess the influence of local secondary structures on the target accessibility, RNAfold [36] was used to calculate the minimum folding energy around the target sites. The results were listed as the supplementary data of predicted targets. The default parameters set in mirTarP include the 7-consecutive base matches as the seed of targeting and the cutoff of mfe of −25 kcal /mol for local dimer formation. The flow chart of mirTarP is illustrated in Figure 1. The advantage of mirTarP over the current prediction tools is that it operates independent of conservation and thus, can be used to find miRNA targets on virus genomes or obtain other interspecies miRNA target predictions. This tool runs quickly and is easy to use with only 2 parameters to be set. Therefore, it will be helpful to wet-lab researchers dealing with new viruses. A comparison of mirTarP to TargetScan and PITA on a dataset of 221 experimentally validated miRNA-target pairs is included in the website along with the tool mirTarP.

The prediction tool mirTarP is free for downloading in the web page.

### 2.3. Prediction of miRNA Targets

By applying mirTarP, 2557 human mature miRNAs were found to have targets in 3133 viral genes, which corresponds to 3376 viral proteins. A total of 181 miRNA records from 13 viral species of 3 families were used for the prediction of targets on human genome. The calculation results showed that these viral miRNAs had potential target sites in 16,439 human genes.

A total of 2,680,194 entries about the miRNAs target sites within human and viral genomes were produced.

## 3. MirTar Database

### 3.1. Web Interface Development

MirTar is designed to adapt a wide variety of screen formats and devices (PCs, tablets, smartphones, etc.). All data were organized by MySQL and the website is implemented in PHP, JavaScript and HTML.

### 3.2. Data Download

**Figure 2:**
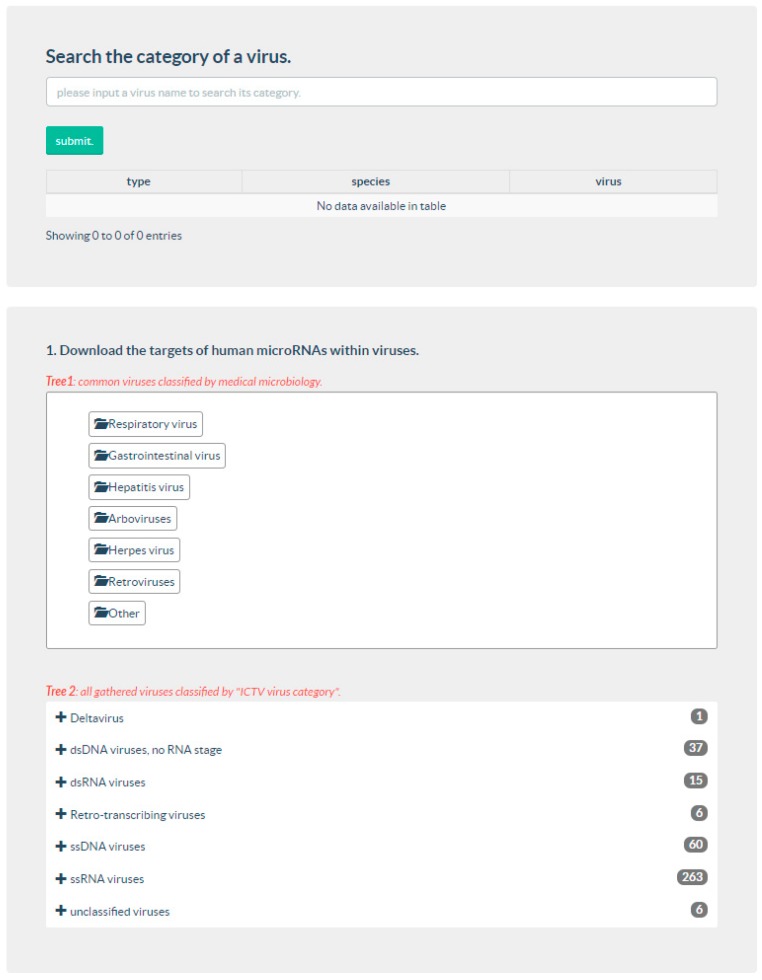
The interface of mirTar database.

The web page provides two ways of data downloads, i.e., customized download and the complete download. The customized download is associated with the items or viruses selected by the user. To provide easy downloading for users from both the molecular biology field and medical field, mirTar database classified the viruses in the following two ways: (1) according to the definition by medical microbiology; and (2) according to ICTV virus category [32]. Currently, the International Committee on the Taxonomy of Viruses (ICTV) provides the most comprehensive, fully annotated compendium of information on virus taxa and taxonomy. Thus, the web page provides a search function for convenient categories when retrieving an input virus. In addition, a python script of the prediction tool mirTarP is available on the web page to facilitate a quick screening of miRNA targets on new viruses. The mirTar database and mirTarP tool are freely available at http://mcube.nju.edu.cn/jwang/mirTar/docs/mirTar/ or http://118.89.139.70/mirTar/docs/mirTar/. The interface of mirTar is shown in Figure 2.

## 4. Conclusions

In this paper, we provide a comprehensive miRNA target database that includes the bidirectional interspecies actions between human and the infectious viruses along with a fast miRNA target prediction program to facilitate a quick screening of miRNA targets on new viruses. The database mirTar contains 2,200,076 candidate target sites on 386 viral genomes for 2577 human mature miRNAs and 480,118 targets of 181 viral mature miRNAs on human genome. The web page of the database was designed for convenient data querying and downloading by classifying the virus species by the two categories of molecular biology and medicine. The database will benefit investigations on the crosstalk between the host and virus gene regulations and the new role of miRNAs in infections and diseases caused by latent viruses, including many cancers.
